# Tailoring cardiovascular risk prediction to females

**DOI:** 10.1530/JOE-25-0084

**Published:** 2025-06-10

**Authors:** Thulani Ashcroft, Marie de Bakker, Atul Anand, Naveed Sattar, Jacqueline A Maybin, Dorien M Kimenai

**Affiliations:** ^1^ British Heart Foundation Centre for Cardiovascular Science, University of Edinburgh, Edinburgh, UK; ^2^ School of Cardiovascular and Metabolic Health, University of Glasgow, Glasgow, UK; ^3^ Centre for Reproductive Health, Institute for Regeneration and Repair, University of Edinburgh, Edinburgh, UK

**Keywords:** female, cardiovascular disease, primary care, risk factors, risk assessment

## Abstract

Atherosclerotic cardiovascular disease (ASCVD) is one of the leading causes of morbidity and mortality in females worldwide. In this review, we provide insights into how sex differences may affect traditional risk factors associated with ASCVD, and give an overview of non-traditional risk factors that have the potential to enhance cardiovascular risk prediction in females. We review clinically applied cardiovascular risk estimation systems, discussing the integration of promising risk factors within these systems. We also explore the role of novel approaches and future directions to refine primary prevention of ASCVD in females. The development of ASCVD varies by sex and age; therefore, cardiovascular risk estimation systems should incorporate both sex and age interactions with risk factors to improve ASCVD risk estimates. As the incidence of non-ASCVD (such as heart failure and arrhythmias) in females continues to rise, it is crucial to adopt a more holistic approach to risk assessment that extends beyond ASCVD outcomes. This review highlights the need for further studies on female-prevalent diseases and female-specific factors that may refine cardiovascular risk estimation in young females. Raising awareness is crucial to ensure studies include individuals from deprived areas and ethnic minorities, as more insights on the intersection between sex and social determinants of health will enhance understanding of the underlying mechanisms of ASCVD risk prevention in females. Finally, taking steps to improve and standardise data on female-specific risk factors throughout a female’s life course could improve preventive cardiovascular care for females.

## Introduction

Cardiovascular disease (CVD), which encapsulates atherosclerotic and non-atherosclerotic outcomes, remains a major global public health challenge and is one of the leading contributors to disability-adjusted life years and death in females worldwide. The estimated age-standardised prevalence and mortality of CVD in females is 6,403 cases per 100,000 (95% confidence interval (CI), 6,079–6,740) and 204 cases per 100,000 (95% CI, 181–222), respectively (Vogel *et al.* 2021, Vervoort *et al.* 2024). The recent State of the Art Review on global burden of cardiovascular disease in women (Vervoort *et al.* 2024), the British Cardiovascular Society (Tayal *et al.* 2024) and the Lancet Women and Cardiovascular Disease Commission (Vogel *et al.* 2021) collectively highlighted the need to address cardiovascular health in females through improving: i) research into sex-specific mechanisms in the pathophysiology of cardiovascular disease, ii) understanding of sex-specific factors that may increase risk, iii) the recognition of the effects of socioeconomic deprivation globally, and iv) interventions that reduce risk of CVD in females.

One individual-level method for reducing cardiovascular risk is the use of cardiovascular risk estimation systems. These systems rely on traditional risk factors to identify high-risk individuals and offer preventative therapies aimed at lowering their risk of future cardiovascular events. However, the performance of these systems has been shown to be suboptimal. Most cardiovascular risk estimation systems have a concordance index of around 0.7, meaning that there is a 70% probability that a person who experiences a cardiovascular event will have a higher risk score than someone who does not (Pennells *et al.* 2019). In a study involving individual participant data from 360,737 individuals, Pennells *et al.* found both over- and under-prediction when testing four commonly used risk estimation systems (Pennells *et al.* 2019). Therefore, novel strategies to improve the accuracy of cardiovascular risk estimation are urgently needed. Given that several female-prevalent and female-specific risk factors have been shown to elevate cardiovascular risk in females, incorporating these factors into risk estimation systems may enhance the accuracy of cardiovascular risk estimates for females.

**Figure 1:**
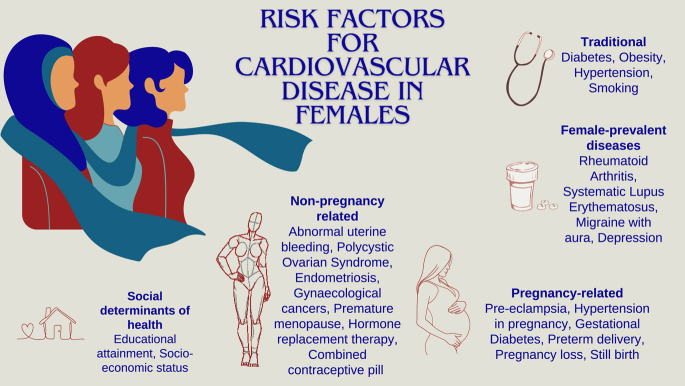
Cardiovascular risk factors in females.

In this review, we provide insights into how sex differences may or may not affect traditional risk factor associations with CVD. We provide an overview of the range of cardiovascular risk factors beyond traditional risk factors that need to be considered in females, from female-prevalent disease risk factors to female-specific risk factors (pregnancy- and non-pregnancy-related), and we discuss the effect of social determinants of health on cardiovascular risk (Fig. 1). We review clinically applied cardiovascular risk estimation systems and discuss the integration of female-specific factors within these systems. Finally, we discuss the role of novel approaches and future directions to refine risk prediction in females in order to prevent CVD. Since this review primarily focuses on biological sex, we use the terms *females* and *males* throughout for clarity.

## Cardiovascular risk factors in females

### Appraisal of research methods

A critical approach is needed when interpreting results of studies examining sex-specific associations with risk factors and CVD. Differences between studies may be attributable to small sample sizes, length of follow-up, methods to control for confounding factors and differences between disease prevalence, care and management across countries. Collider bias is also important to consider when assessing sex-specific associations between risk factors and CVD using observational data. This bias occurs when the exposure and outcome are both associated with another variable included in the analysis. For example, sex and age are both independent risk factors for CVD. When only older individuals with CVD are selected, this may introduce a spurious association between sex and CVD. An additional consideration is that studies often use different definitions for their composite endpoint of CVD. In this review, we use the term CVD for studies that include non-atherosclerotic heart disease, such as heart failure and arrhythmia, in their composite outcome; and for the studies that specify only atherosclerotic disease (e.g. ischaemic heart disease), we use the term ASCVD.

### Traditional risk factors

#### Blood pressure

Despite these caveats, there is mounting evidence that there are differences in the development of CVD between females and males. Females are at lower absolute risk for CVD compared to males, with females experiencing their first CVD event on average a decade later than males (Bergström *et al.* 2021). However, the progression of CVD accelerates as females age. We have previously found that cardiac troponin concentrations – the gold standard biomarker for myocardial injury – are different in females and males. Cardiac troponin concentrations are persistently lower in females as compared with males throughout the life course. However, cardiac troponin increases with age in both sexes, and the relative increase of cardiac troponin trajectories is steeper in females than in males over time (de Bakker *et al.* 2023). Sex-related differences have also been observed in systolic blood pressure trajectories, with females reaching the same values as males around midlife, and the increase in systolic blood pressure from baseline showing a steeper gradient in females than in males with increasing age (Ji *et al.* 2020). The underlying pathophysiological mechanisms of these observations are still not well understood. Importantly, the lower baseline CVD risk in females also contributes to the greater relative risk observed in females.

#### Diabetes and obesity

For diabetes, the absolute risk gain for CVD from type 2 diabetes is similar for females and males, but the relative risk for type 2 diabetes is higher for females than males (Sattar 2019). The higher relative risk of CVD associated with type 2 diabetes in females as compared to males is likely due to differences in body composition. Females have the capacity to carry more subcutaneous fat and have less visceral fat than males (Sattar & Gill 2014). This in part explains why females have to put on more weight than males overall to overcome this protective body fat distribution to develop diabetes.

When females are diagnosed with type 2 diabetes, the adjusted mean difference in body mass index between those with and without type 2 diabetes is higher in females than in males (in 20–39 years age group, adjusted mean difference in body mass index for those with and without type 2 diabetes: females 8.6 (95% CI, 8.4–8.9) and males 5.2 (95% CI, 5.0–5.3)) (Wright *et al.* 2020). A similar effect was seen for systolic blood pressure, with adjusted mean difference being higher for females than males (in 20–39 years age group, adjusted mean difference of systolic blood pressure between those with and without type 2 diabetes: females 8 (95% CI, 7–9); for males 6 (95% CI, 5–7)) (Wright *et al.* 2020). In this UK-based study, the mean differences in body mass index, weight and systolic pressure between those diagnosed with type 2 diabetes and those without decreased with increasing age in both sexes. A study using the Swedish National Diabetes Registry found comparable results, with the highest excess risk of ASCVD in those with type 2 diabetes vs those without, being in females aged 40 years or under (Sattar *et al.* 2019). This highlights the importance of identifying and managing these risk factors in these young populations.

#### Smoking

Previous studies also showed a modifying effect of sex on the relationship between smoking status and CVD, with a meta-analysis revealing the pooled adjusted female-to-male relative risk ratio of smoking vs non-smoking for ASCVD was 1.25 (95% CI, 1.12–1.39) (Huxley & Woodward 2011). These findings may be influenced by the fact that females are more likely to underreport their smoking behaviour compared to males but more evidence is required to explain the observed sex differences.

#### Clinical translation of observed sex differences in traditional risk factors

The development of CVD varies by sex and age, therefore cardiovascular risk estimation systems should incorporate both sex and age interactions with traditional risk factors to improve ASCVD risk estimates. When examining traditional risk factor reduction, the use of antihypertensive, antidiabetic and lipid-lowering medications shows large reductions in CVD events and appears equally effective in both sexes (Brugts *et al.* 2009, Bidel *et al.* 2023, Hanlon *et al.* 2025). This highlights the importance of providing females and males with preventative treatment when they are identified as high risk through commonly applied cardiovascular risk estimation systems that include traditional risk factors.

### Female-prevalent disease risk factors

Cardiovascular disease has been linked with several diseases that are more prevalent in females than males, e.g. the autoimmune diseases rheumatoid arthritis and systemic lupus erythematosus (Conrad *et al.* 2023) and migraine with aura (Kalkman *et al.* 2023). Depression is also more common in females than males and depression diagnosed during pregnancy has been associated with CVD, even after adjusting for traditional and pregnancy-related risk factors. A Swedish study reported a higher relative risk of CVD (adjusted hazard ratio (aHR) 1.36 (95% CI, 1.31–1.42)) for females with depression ascertained during pregnancy and up to 12 months postpartum compared to pregnant females without depression (Lu *et al.* 2024). A study from the United States (US) also reported a higher risk of ischaemic heart disease (aHR 1.83 (95% CI, 1.20–2.80)) in those with depression diagnosed between 6 weeks gestation and delivery date vs pregnant females without a depression diagnosis (Ackerman-Banks *et al.* 2023). There may also be differences towards the end of the life course as females have higher prevalence of Alzheimer’s disease (Moutinho 2025), multimorbidity (Chowdhury *et al.* 2023) and frailty than males (James *et al.* 2024). The impact of these on cardiovascular outcomes is more complex as they often share risk factors with ASCVD and can have a bidirectional relationship with ASCVD.

Overall, though ASCVD has higher prevalence overall in males than females, there are conditions which are associated with ASCVD which are more common in females (e.g. rheumatoid arthritis). It is unclear if there is a greater cardiovascular impact from these conditions in females than males, such as effects of medications, or whether the weighting of risk factors between females and males may be somewhat different. Nonetheless, they highlight the need to investigate conditions which arise predominantly in females as a potential mechanism to improve the precision of cardiovascular risk estimates in females.

### Female-specific risk factors

#### Pregnancy-related risk factors

Females are most likely to be in regular contact with healthcare services during pregnancy with visits to primary care, midwifery and obstetric services. Therefore, most research into female-specific risk factors has focussed on obstetric outcomes. Gestational diabetes and hypertensive disorders of pregnancy, such as hypertension in pregnancy and pre-eclampsia, are well known to be associated with greater ASCVD risk (Tschiderer *et al.* 2023). Higher ASCVD risk has also been linked with preterm delivery, pregnancy loss and stillbirth (Tschiderer *et al.* 2023). All these conditions can be collectively referred to as adverse pregnancy outcomes and prevalence was found to be 30% of all pregnancies in a recent Swedish cohort study (Crump *et al.* 2024). As pregnancy serves as a metabolic and vascular stress test, adverse pregnancy outcomes may be metabolic or vascular indicators for future ASCVD (Sattar & Greer 2002), with the risk of ASCVD mediated by ‘upstream’ traditional cardiovascular risk factors such as dyslipidaemia, diabetes, obesity and hypertension. Despite awareness of potential future cardiovascular risk from conditions such as gestational diabetes and established recording of adverse pregnancy outcomes in health records, a recent Scientific Statement by the American Heart Association highlighted the lack of evidence-based post-pregnancy interventions to reduce long term risk in these females (Lewey *et al.* 2024).

#### Gynaecological symptoms as risk factors

Most of a female’s life course is not associated with pregnancy and an increasing number of females remain childless. In comparison to pregnancy-related factors, gynaecological symptoms and conditions have been understudied. Menstrual bleeding which is abnormal in volume, regularity and/or timing is common, affecting up to a third of females (Liu *et al.* 2007). The true prevalence is widely understood to be higher than reported levels due to social taboos and normalisation of symptoms (Munro *et al.* 2018). Abnormal uterine bleeding includes prolonged menstrual bleeding, heavy menstrual bleeding, irregular menstruation and menstruations that are too frequent, infrequent or intermenstrual bleeding (Munro *et al.* 2018). These menstrual symptoms are not routinely collected in medical records, despite 50% of the female population experiencing issues with menstruation during their reproductive years. The association of menstrual symptoms with CVD is an emerging area of research and, as with other observational studies, there is heterogeneity with regards to measurement, definitions of exposures and outcomes and methodological approaches. The currently available evidence indicates that females with abnormal uterine bleeding may be at increased CVD risk when compared to females with normal menstrual bleeding parameters (Table 1). Of the few studies conducted, most seem to have focussed on the regularity and frequency of cycles but have described the exposure in various ways, from cycle length, irregular alone or grouped with irregular and no periods or combined irregular and longer periods. In general, they all appear to suggest an association with CVD but the definition for cardiovascular event and inclusion of confounding factors varies by study. Only one cross-sectional study examined heavy menstrual bleeding, reporting higher odds of a major cardiovascular event in those with heavy menstrual bleeding vs those without heavy menstrual bleeding (females 40 years and younger, OR 1.61 (95% CI, 1.25–2.08); females over 40 years, OR 1.19 (95% CI, 1.10–1.42)) (Dubey *et al.* 2024).

**Table 1:** Studies investigating menstrual symptoms and cardiovascular risk.

Author, year	Study design	Exposure (s)	Comparator	Definition of cardiovascular event	Fully adjusted effect estimates (95% CI)	Variables in fully adjusted model
Okoth *et al.* (2023)	Cohort	Irregular or frequent and infrequent menstrual cycles	Regular menstrual cycles	IHD, heart failure, cerebrovascular disease. Estimates were also provided for each condition separately	Frequent or infrequent: HR 1.24 (95% CI, 1.08–1.52) irregular: HR 1.08 (95% CI, 1.00–1.19)	Age, area-based deprivation, BMI, smoking, lipid medication, alcohol status, HTN, DM, CHCP, connective tissue disorders, migraine, PCOS, GM, pre-eclampsia, pre-term birth, PID, endometriosis, fibroids
Huang *et al.* (2023)	Cohort	Irregular menstrual cycle length	Regular cycle length	Fatal and nonfatal MI, AF and cardiac arrhythmias, heart failure, cerebrovascular disease. Estimates were also provided for each condition separately	Short cycle length: HR 1.29 (95% CI, 1.11–1.50) long cycle length HR: 1.11 (95% CI, 0.98–1.56)	Age, education, race or ethnicity, HTN, DM, cholesterol, SHBG, menarche, family of CVD or stroke, CHCP, depression. Model 3 further adjusted BMI, smoking, alcohol, physical activity
Wang *et al.* (2022)	Cohort	Irregular or no periods and longer or too irregular to estimate, at various time points	Regular cycles	Fatal and nonfatal MI, CHD, coronary revascularization, stroke	Irregular or no periods: 14–17 years, HR 1.15 (95% CI, 0.99–1.34); 18–22 years, HR 1.36 (95% CI, 1.06–1.75); 29–46 years, HR 1.40 (95% CI, 1.14–1.71) longer or too irregular: 18–22 years, HR 1.44 (95% CI, 1.13–1.84); 29–46 years, HR 1.30 (95% CI, 1.09–1.57)	Age, age at menarche, race and ethnicity, FH of CVD, BMI, menopausal status, HRT, parity, physical activity, smoking, diet
Dubey *et al.* (2024)	Cross-sectional	Heavy menstrual bleeding (excessive and heavy bleeding both regular and irregular cycles)	No heavy menstrual bleeding	Major adverse cardiovascular event which is any event related to stroke, MI and heart failure	OR 1.61 (95% CI, 1.25–2.08) in those ≤40 years old OR 1.19 (95% CI, 1.10–1.42) in those >40 years old	Propensity score matched analysis. Adjusted for race/ethnicity, age, household income, primary payers, smoker, alcohol, CHCP, metabolic syndrome, NSAIDs, leiomyoma uterus, obesity, PCOS, anaemia, anticoagulants

CI, confidence interval; HR, hazard ratio; OR, odds ratio; IHD, ischaemic heart disease; MI, myocardial infarction; AF, atrial fibrillation; DM, diabetes mellitus; HTN, hypertension; CVD, cardiovascular disease; BMI, body mass index; CHCP, combined hormonal contraceptive pill; PCOS, polycystic ovarian syndrome; GM, gestational diabetes; PID, pelvic inflammatory disease; SHBG, sex hormone-binding globulin; HRT, hormone replacement therapy; NSAIDs, nonsteroidal anti-inflammatory drugs.

#### Gynaecological conditions as risk factors

Abnormal uterine bleeding is a symptom and underlying causes include structural conditions including endometrial polyps, uterine fibroids or adenomyosis, or non-structural causes such as coagulation disorders, ovulatory dysfunction (e.g. polycystic ovarian syndrome) or aberrations in endometrial function (Munro *et al.* 2018). The evidence for gynaecological conditions being associated with ASCVD is understudied and remains unclear. One small study (Coronary Artery Risk Development in Young Adults Study, *n* = 972) did not find an association between fibroids (non-cancerous tumours of the myometrium) and subclinical CVD (adjusted odds ratio (aOR) using coronary artery calcification, carotid intima media thickness and left ventricular mass as outcomes), despite traditional cardiovascular risk factors being more common in those with fibroids (Laughlin-Tommaso *et al.* 2019). However, a larger cross-sectional study (*n* = 5,552) reported higher odds of ASCVD in those with fibroids vs those without (aHR 1.60 (95% CI, 1.08–2.37)) (Brewster *et al.* 2022). Polycystic ovary syndrome, a heterogeneous reproductive endocrine disorder, can cause problematic menstruation and other symptoms including hirsutism, acne and infertility. A nationwide Danish study found a higher ASCVD event rate in those with polycystic ovary syndrome than controls (22.6 per 1,000 vs 13.6 per 1,000) over their 11-year study period. In addition, obesity, diabetes and infertility increased the risk of developing ASCVD in those with polycystic ovary syndrome (Glintborg *et al.* 2018). However, evidence from observational studies on association between polycystic ovary syndrome and ASCVD is conflicting, with some studies finding no association after adjusting for traditional risk factors (Osibogun *et al.* 2020). This may be due to difficulty unravelling the underlying mechanisms, as polycystic ovary syndrome is closely associated with abnormal lipids, obesity and impaired glucose tolerance, all of which independently contribute to increased cardiovascular risk. It may also be related to flaws in study design such as insufficient length of follow-up and age of participants. Endometriosis is a prevalent condition in reproductive-aged females where tissue similar to the endometrium is found in ectopic locations, often presenting with pain. Studies suggest that those with endometriosis may have a higher risk of ASCVD (Blom *et al.* 2023, Tripathi *et al.* 2024), but designing such studies is difficult as endometriosis has a delayed diagnosis of approximately 8 years, making identification of cases and controls problematic. There are also challenges in establishing links with ASCVD and gynaecological (cervical, ovarian and endometrial) cancers. Risk factors such as obesity can be more prevalent in these patients and treatments may involve cardiotoxic chemotherapy, making it difficult to determine how much of the risk is attributable to the cancer itself (Tripathi *et al.* 2024).

#### Management of gynaecological symptoms as risk factors

The management of gynaecological symptoms and conditions also requires consideration when examining risk prediction for CVD. A large study of pooled individual-level patient data (*n* = 301,438) showed that ASCVD risk is greater in those with a lower age of menopause (Zhu *et al.* 2019). Premature menopause (before 40 years) had the highest risk, followed by early menopause (40–44 years) (premature menopause aHR 1.55 (95% CI, 1.38–1.73), early menopause aHR 1.30 (95% CI, 1.22–1.39)) when compared to menopause at 50–51 years (Zhu *et al.* 2019). Given this, it is possible that surgical interventions that cause premature menopause, i.e. bilateral oophorectomy before 50–51 years, may be associated with higher ASCVD. Using the Nurses’ Health Study cohort data, (Farland *et al.* 2023) reported a higher risk of ASCVD in those who underwent hysterectomy, with and without oophorectomy, compared to those who did not have surgery (aHR hysterectomy: HR 1.19 (95% CI, 1.02–1.39), HR hysterectomy with unilateral oophorectomy: 1.40 (95% CI, 1.08–1.82) and HR hysterectomy with bilateral oophorectomy: 1.27 (95% CI, 1.07–1.51)) (Farland *et al.* 2023). Importantly, this study found the risk was greatest in those who were 50 years or younger at the time of surgery and that ASCVD risk was increased even in those whose ovarian function was preserved. Unfortunately, this study did not report the indication for surgery; therefore, it is unclear if their results were due to the treatment itself, the underlying condition or if those with heavy menstrual bleeding have some underlying dysfunction that contributes to ASCVD. In addition, participants were relatively young, with a mean age below 40 years (Farland *et al.* 2023).

Cardiovascular risk due to hormonal treatment for menopause and contraception has also been examined. In postmenopausal women, the long-term effect of taking hormone replacement therapy as oestrogen alone or with progesterone was reviewed for the US Preventive Services Task Force in 2022 (Gartlehner *et al.* 2022). The authors found an absolute risk increase for stroke for oestrogen alone and combination oestrogen and progesterone compared to placebo (79 more cases per 10,000 persons (95% CI, 15–159) over 7.2 years for oestrogen vs placebo, 52 more cases per 10,000 persons (95% CI, 12–104) over 5.6 years for oestrogen and progesterone combined). No association was found with coronary heart disease for oestrogen alone or with progesterone. For the combined hormonal contraceptive pill (oestrogen and progestogen), a Cochrane review found an increased risk of both ischaemic heart disease (relative risk (RR) 1.6 (95% CI, 1.2–2.1)) and ischaemic stroke (RR 1.7 (95% CI, 1.5–1.9)) (Roach *et al.* 2015) such that there are specific clinical guidelines for use of these contraceptives.

### The effect of social determinants of health on cardiovascular risk

The socioeconomic gradient for ASCVD is well established (Backholer *et al.* 2017, Peters *et al.* 2020, Conrad *et al.* 2024) and several traditional, female-prevalent disease and female-specific risk factors are more prevalent in areas with greatest social deprivation. It has been suggested that females may be more vulnerable to the effects of socioeconomic deprivation than males because of differences in gender pay, increased likelihood of single parenting and, in some countries, due to lower education (Vogel *et al.* 2021, Vervoort *et al.* 2024). A large United Kingdom (UK) study created sex-specific models for cardiovascular risk estimation (QR4) and found a higher relative risk of ASCVD associated with area-based deprivation for their female model than the model created for males (aHR 1.26 (95% CI, 1.22–1.29) for females, aHR 1.11 (95% CI, 1.09–1.13) for males) (Hippisley-Cox *et al.* 2024). However, a more nuanced approach is required when understanding any sex differences by socioeconomic status as there can be differences based on the measure and the outcome. For example, a meta-analysis of multiple cohort studies reported higher stroke risk for males of lower educational attainment compared to higher educational attainment than the risk seen in females (age-adjusted HR 1.34 (95% CI, 1.07–1.69) for females, age-adjusted HR 1.53 (95% CI, 1.27–1.89) for males), but the reverse was found with coronary heart disease (age-adjusted HR 1.66 (95% CI, 1.46–1.88) for females, age-adjusted HR 1.30 (95% CI, 1.15–1.48) for males) (Backholer *et al.* 2017). Irrespective of sex, socioeconomic status is important and the recently published 2024 European Society of Cardiology Guidelines for the management of elevated blood pressure and hypertension recognise socioeconomic status as a non-traditional risk modifier (McEvoy *et al.* 2024).

### Ethnicity

Differences in the prevalence of ASCVD have been observed across various ethnic groups, particularly in studies conducted in the US and UK (Ho *et al.* 2022, Post *et al.* 2022). However, it is important to recognise the contribution of other factors to these disparities, as the effects of ethnicity and socioeconomic position are likely intertwined. For example, a study by Shah *et al.* showed that population-level differences in ASCVD prevalence across ethnic groups in the US were due to several underlying social factors, with major contributors being education and place of birth (Shah *et al.* 2023). Moreover, the Multi-Ethnic Study of Atherosclerosis (MESA) study reported a 72% higher relative risk of cardiovascular mortality in those of Black heritage vs White individuals (HR 1.72 (95% CI, 1.34–2.21)) (Post *et al.* 2022). However, the risk was attenuated after adjusting for socioeconomic position (HR 1.37 (95% CI, 1.02–1.84)) (Post *et al.* 2022). Social factors were also found to be drivers for ethnic difference in maternal cardiovascular health in the US. A study on first-trimester nulliparous females found educational attainment explained the greatest proportion of difference in cardiovascular health between Hispanic vs non-Hispanic White and non-Hispanic Black vs non-Hispanic White females (Cameron *et al.* 2025). Similarly, a study using UK Biobank data reported that South Asian (HR 1.69 (95% CI, 1.59–1.79)) and Black (HR 1.12 (95% CI, 1.03–1.22)) participants had a higher risk of ASCVD vs White participants (Ho *et al.* 2022). Adjusting for social deprivation removed the ASCVD risk disparity for Black participants (HR 0.90 (95% CI, 0.82–0.98)) but only attenuated the risk among South Asians (HR 1.55 (95% CI, 1.45–1.64)) (Ho *et al.* 2022). The risk attributable to adiposity was highlighted in this study; adiposity affected all ethnic groups but contributed to a higher proportion of cases from Black and South Asian ethnic minorities.

To reduce the risk of ASCVD in the most vulnerable females, such as those in deprived areas or from specific ethnic groups, it is important to untangle the main drivers of their increased ASCVD risk. Engagement with health services and medical research may also be different in these populations, with mistrust and fear of discrimination among other factors leading to ethnic inequalities (Kapadia *et al.* 2022). In addition, recording of ethnicity in health records is often either poorly done or inadequate, as historical migration patterns reflect the options for coding of ethnicity in health records (Pineda-Moncusí *et al.* 2024). The accuracy of cardiovascular risk estimation systems in predicting future CVD in these underserved communities depends on improved coding with education of healthcare professionals and, in part, on the diversity of the derivation cohort used to develop the risk score. Studies that include individuals from deprived areas and ethnic minorities are essential to ensure accurate cardiovascular risk estimation across all subgroups.

### Transgender

Another minority group are those who are transgender. This review has focussed on biological sex rather than gender, with the term female applied to those of female sex. Research on transgender individuals is in its infancy. A recent systematic review and meta-analysis has suggested an increased risk of stroke in transgender women vs cisgender men (pooled RR 1.3 (95% CI, 1.0–1.8)) and transgender men compared to cisgender women (pooled RR 1.3 (95% CI, 1.0–1.6)) (van Zijverden *et al.* 2024). Although no association was seen with myocardial infarction, the study emphasises the need for further research in this under-recognised and understudied area.

## Cardiovascular risk estimation systems to predict CVD risk

### Clinically applied cardiovascular risk estimation systems

The use of a cardiovascular risk estimation system is integral to the primary prevention of CVD. A derivation cohort that includes individuals free from cardiovascular disease at baseline is used for model development. Current cardiovascular risk estimation systems are developed using data from electronic health records, observational cohort(s) or a combination of both. Ideally, contemporary target population data from the country or region are used to optimise performance in common practice. All clinically applied risk systems are sex-specific and include risk factors chosen by developers to further optimise model performance (Table 2). Cardiovascular risk estimation systems are usually externally validated on an independent dataset, based on a population with similar characteristics.

**Table 2:** Risk factors included in clinically applied cardiovascular risk estimation systems for females.

	QR4	ASSIGN V.2.0	SCORE2	SCORE2-OP	Prevent	Reynolds
Author, year	Hippisley-Cox (2024)	Welsh (2025)	SCORE2 Working Group and ESC Cardiovascular Risk Collaboration 2021	SCORE2-OP Working Group and ESC Cardiovascular Risk Collaboration 2021	Khan (2024)	Ridker (2007)
Region	UK	Scotland	Europe	Europe	US	US
For female within age range	18–84	30–74	40–69	70–89	30–79	45 years or older
Included age interactions	Yes	No	No	No	Yes	No
Traditional risk factors	Age, DM, BMI, cholesterol (total/HDL ratio), SBP, smoking, family history of CVD	Age, DM, cholesterol (total, HDL), SBP, smoking, family history of CVD	Age, cholesterol (total, HDL, LDL), SBP, smoking	Age, cholesterol (total, HDL, LDL), SBP, smoking	Age, DM, BMI, cholesterol (total, HDL, LDL), SBP, smoking	Age, DM, SBP, cholesterol (total, HDL) smoking
Disease-specific risk factors	Learning disability, down syndrome, rheumatoid arthritis, atrial fibrillation, renal failure, migraine, systemic lupus, severe mental illness, chronic obstructive lung disease, lung cancer, oral cancer, blood cancer, brain cancer	None	None	None	None	None
Social determinant of health	Social deprivation	Social deprivation	None	None	Social deprivation	None
Sex-specific risk factors	Postnatal depression, pre-eclampsia	None	None	None	None	None
Other risk factors	Ethnicity, corticosteroids, atypical antipsychotics	None	Risk Region in Europe	Risk Region in Europe	Antihypertensive medication, lipid lowering medication, eGFR, HbA1c, urine ACR	High sensitivity CRP
Outcomes	Fatal and non-fatal IHD, TIA, cerebrovascular disease	CHD, stroke, TIA, carotid endarterectomy, CABG, carotid angioplasty, percutaneous transluminal coronary angioplasty, deaths from cardiovascular causes	Hypertensive disease, IHD, arrhythmias, heart failure, cerebrovascular disease, atherosclerosis sudden death within 24 h of symptom onset, non-fatal MI and stroke	Hypertensive disease, IHD, arrhythmias, heart failure, cerebrovascular disease, atherosclerosis sudden death within 24 h of symptom onset, non-fatal MI and stroke	Fatal and non-fatal ASCVD (CHD, MI and stroke) and heart failure	MI, ischaemic stroke, coronary revascularisation, cardiovascular deaths

DM, diabetes mellitus; SBP, systolic blood pressure; BMI, body mass index; eGFR, estimated glomerular filtration rate; HbA1c, haemoglobin A1c; ACR, albumin-creatinine ratio; CRP, C-reactive protein; MI, myocardial infarction; IHD, ischaemic heart disease; TIA, transient ischaemic attack; CHD, coronary heart disease; CABG, coronary artery bypass graft; AAA, abdominal aortic aneurysm.

Commonly applied cardiovascular risk estimation systems for females are: QR4 (Hippisley-Cox *et al.* 2024), ASSIGN V.2.0 (Welsh *et al.* 2025), SCORE2 (SCORE2 Working Group and ESC Cardiovascular Risk Collaboration 2021), SCORE2-OP (SCORE2-OP Working Group and ESC Cardiovascular Risk Collaboration 2021), PREVENT (Khan *et al.* 2024) and Reynolds (Ridker *et al.* 2007). Differences in characteristics between derivation cohorts and selection of risk factors included in the models do exist (Tables 2 and 3). QR4 and ASSIGN V.2.0 are developed and intended for use in the UK and Scottish populations, respectively. PREVENT and Reynolds are based on observational data from the US, and SCORE2 and SCORE2-OP were developed for use across Europe. SCORE2 and SCORE2-OP attempted to account for differences in baseline cardiovascular risk between countries by categorising them into low, medium, high and very high-risk regions, based on World Health Organisation ASCVD mortality data.

**Table 3:** Cohort characteristics of clinically applied cardiovascular risk estimation systems.

	QR4	ASSIGN 2	SCORE2	SCORE2-OP	Prevent	Reynolds
**Cohort for derivation**						
Author, year	Hippisley-Cox (2024)	Welsh (2025)	SCORE2 Working Group and ESC Cardiovascular Risk Collaboration 2021	SCORE2-OP Working Group and ESC Cardiovascular Risk Collaboration 2021	Khan (2024)	Ridker (2007)
Data source	Electronic health records: English (QResearch derivation)	Generation Scotland Scottish Family Health Study, UK Biobank	45 cohorts in Denmark, France, Germany, Greece, Italy, Netherlands, Norway, UK, Spain, Sweden, US and Canada	Connor study in Norway: Included individuals 65 years or older	General population cohorts in US; ARIA, CARDIA, CHS, FHS, JHS, MESA and EHR Geisinger Health and Optum Labs Data Warehouse (electronic health records)	Female’s Health Study (US Female study)
Size (*n*)	9,976,306	44,947	677,684	28,503	3,281,919	16,400
Female (%)	51.7	57.7	66	50	56	100
Age (years)	Mean 39.0 (SD, 15.0)	Mean 55 (SD, 8)	Mean 57 (SD, 9)	Mean 73 (SD, 5)	Mean 53 (SD, 13)	Median 52 (IQR, 48–58)
Ethnicity (%)	Recorded in 62% of records	Not reported	Not reported	Not reported	White: 78	White: 95.2
	White: 44				Black: 9	Black: 1.9
	Asian: 8.3				Hispanic: 5.7	Hispanic: 1
	Black: 2.9				Asian: 2.6	Asian: 1.4
	Chinese: 1.5				Other and missing: 4	Other: 0.5
	Other: 4.4					
Time period	Data from 2010 to 2021	Recruitment period 2006–2010	Recruitment period 1990–2009, end date varied based on cohort	Recruitment period 1994–2003, end date varied based on cohort	Data from 1992 (2008 start date for EHR) to 2021	1992–2004
Follow up	Proportion with 10 years or more 18.9%	10 years	Median 10.7 years (IQR, 5.0–18.6)	Median 13 (IQR, 8–15)	Mean 4.8 (SD, 3.1), follow up censored at 15 years	Median 10.2 (IQR, 9.7–10.6)
**Cohort for validation**						
Data source	English (QResearch validation) and CPRD Scotland Wales Northern Ireland	UK Biobank	25 cohorts: Denmark, France, Norway, Spain, Netherlands, UK, Finland, Germany, Italy, Sweden, Czech Republic, Estonia, Poland, Lithuania, Russia	Six cohorts: three in US (included MESA), two in UK, one in Europe	General population cohorts: REGARDS, CRIC, RBS and electronic health records Optum Labs Data Warehouse	Female’s Health Study
Size (*n*)	English 3,246,602; CPRD 3,54,007	436,796	1,133,181	338,615	3,330,085	8,158
Female (%)	English 51.8; CPRD 52	44.3	50.9	57.8	57	100
Age (years)	English 38.9 (14.9); CPRD 42.6 (16.4)	56.2 (8.1)	53.2 (9.5)	74.1 (6)	52 (13)	Median 52 (49–59)
Ethnicity (%)	English (recorded in 60.7%):	Not reported	Not reported	Not reported	White 79	White 95.3
	White 42.9				Black 9.4	Black 1.9
	Asian 6.6				Hispanic 4.0	Hispanic 1
	Black 2.9				Asian 2.5	Asian 1.5
	Chinese 1.5				Other and missing 5.2	Other 0.3
	Other 4.5					
	CPRD (recorded in 35.5%):					
	White 32.6					
	Asian 1.2					
	Black 0.4					
	Other 0.8					
Validation finding	C-statistic in females in England 0.864 (95% CI, 0.862–0.866); wales 0.829 (95% CI, 0.825–0.832); Scotland 0.837 (95% CI, 0.834–0.839), Northern Ireland 0.847 (95% CI, 0.841–0.853). Also validated in ethnic groups	C-statistic in females 0.706 (95% CI, 0.702–0.710)	C-statistic applied to all validation cohorts results ranged from 0.67 (0.65–0.68) to 0.81 (0.76–0.86). Not specific for females	C-statistics ranged from 0.63 (0.61–0.65) to 0.67 (0.64–0.69). Not specific for females	C-statistic for female 0.794 (IQR 0.763–0.809). Also validated in ethnic groups	C-statistic 0.809 (no range given)

ARIA, Atherosclerosis Risk in Communities; CARDIA, Coronary Artery Risk Development in Young Adults; CHS, Cardiovascular Health Study; FHS, Framingham Heart Study; JHS, Jackson Heart Study; MESA, Multi-Ethnic Study of Atherosclerosis; REGARDS, Reasons for Geographic and Racial Differences in Stroke; CRIC, Chronic Renal Insufficiency Cohort; RBS, Rancho Bernardo Study; CPRD, Clinical Practice Research Datalink; IQR, interquartile range; SD, standard deviation.

With regards to risk factors, diabetes is not included in SCORE2 or SCORE2-OP, as a specific tool for people with diabetes has been developed (SCORE2-Diabetes) (SCORE2-Diabetes Working Group and the ESC Cardiovascular Risk Collaboration 2023). The QR4 risk model is the only model that includes body mass index. PREVENT, ASSIGN V.2.0 and QR4 include social deprivation status, with QR4 also adding ethnicity to the risk score. The developers of PREVENT decided not to include ethnicity as a predictor for ASCVD, reasoning that differences associated with ethnicity are primarily driven by social determinants of health rather than ethnicity itself. PREVENT does include antihypertensive and lipid-lowering medication as predictors for ASCVD risk, as well as laboratory parameters for renal function and diabetes. The Reynolds risk score includes C-reactive protein measurements. QR4 includes corticosteroids, antipsychotic medications, an additional 13 disease-specific risk factors and is the first and only tool to include the female-specific risk factors pre-eclampsia and postnatal depression (Hippisley-Cox *et al.* 2024).

Another key difference between risk systems is how they incorporate the effect of age on risk factors and the selection of CVD outcomes. Both QR4 and PREVENT incorporate age interactions in their risk models, whereas SCORE2 and SCORE2-OP are age-specific models designed for younger and older individuals, respectively. Although all risk scores include fatal and non-fatal ischaemic heart disease, coronary artery disease, myocardial infarction, stroke and cerebrovascular disease, some also account for hypertensive disease and abdominal aortic aneurysms (SCORE2, SCORE2-OP), non-atherosclerotic diseases such as heart failure (SCORE2, SCORE2-OP, PREVENT), arrhythmias (SCORE2, SCORE2-OP) and interventions such as coronary angioplasty (ASSIGN V.2.0, Reynolds). The incidence of non-atherosclerotic disease in females is rising (Conrad *et al.* 2024), therefore it is crucial to adopt a more holistic approach to risk assessment that extends beyond ASCVD outcomes to other CVD.

### Inclusion of female-specific risk factors in cardiovascular risk estimation systems

(Tschiderer *et al.* 2023) reviewed nine studies that incorporated female-specific factors such as hypertensive disorders of pregnancy and gestational diabetes into risk estimation systems for primary prevention of CVD. Of these, only one study included factors unrelated to pregnancy, such as age at menarche or menopause, menopausal status and hormone replacement therapy. In addition, a recent UK Biobank study explored 13 female-specific factors, six of which were non-pregnancy related (Doust *et al.* 2024). In all these studies, the addition of female-specific factors resulted in little or no improvement in model discrimination or reclassification. One possible reason for this could be that some female-specific factors, particularly those related to pregnancy, are closely linked to traditional risk factors or may be heightened by them. As a result, the inclusion of traditional risk factors might already capture the increased ASCVD risk associated with certain female-specific factors. Another explanation could relate to the characteristics of the derivation cohorts used to develop the models. Most risk estimation systems were developed using cohorts of older females, with mean ages typically ranging from 49 to 53 years (Table 3), while the UK Biobank study included a slightly older group with a median age of 58 years (Doust *et al.* 2024). However, female-specific risk factors are more prevalent in younger women; as a result, the data used to build existing models may not adequately capture these factors. QR4 is the only risk estimation system that used a younger population for its derivation cohort, with a mean age of 39 years. This tool was developed for a broader population aged 18–84 years, whereas other models are intended for those aged 30 and older. With the largest sample size for model derivation – 9,976,306 participants – QR4 may offer more robust insights. In contrast, the relatively small sample sizes in other studies could explain the lack of improvement in model performance observed elsewhere. Given these uncertainties, external validation of QR4 in diverse populations is crucial for enhancing our understanding of female-specific risk factors and improving CVD risk prediction. Finally, while metrics such as discrimination, reclassification and calibration offer valuable insights into model performance at population level, they do not fully capture the clinical value necessary to determine whether incorporating female-specific factors can clinically meaningfully impact the prevention of CVD in females. Further research is needed to assess the clinical added value of including female-specific factors in risk models, with a focus on metrics that extend beyond traditional performance indicators (e.g. decision curve analysis).

### Novel strategies to enhance cardiovascular risk estimation systems for females

Multiple strategies have been proposed to enhance cardiovascular risk prediction, such as the addition of cardiac biomarkers or the development of polygenic risk scores. Recent data on cardiac biomarkers support N-terminal pro-B-type natriuretic peptide, growth differentiation factor-15 and cardiac troponins as the most promising biomarkers for refining cardiovascular risk estimates in both females and males (Welsh *et al.* 2023). However, further research is needed to evaluate the clinical impact of using risk assessment tools that include cardiac biomarkers. The combination of a polygenic risk score with the QRISK2 risk estimation system has shown promising results, identifying a greater proportion of individuals who went on to have a major cardiovascular event with the combined system compared to QRISK2 alone (Samani *et al.* 2024). This additive approach appeared to perform particularly well in younger individuals, with a strong relative increase in those aged between 40 and 54 years, and was similarly beneficial for both females and males.

With the introduction of more advanced statistical modelling, such as machine learning, it has become feasible to incorporate more complex modalities (e.g. electrocardiograms and cardiac imaging) and large quantities of routinely collected healthcare data into cardiovascular risk estimation systems (Adedinsewo *et al.* 2022). Machine learning approaches offer the potential for more accurate risk estimation compared to standard statistical methods, as they can analyse large, complex datasets to identify patterns that may indicate a higher future risk of ASCVD. However, using data that lack information on female or minority groups, or suffer from poor-quality female-relevant healthcare data, may exacerbate existing inequalities for females. Machine learning cannot overcome these deficiencies, and the creation of ‘black box’ algorithms makes it difficult to detect biases, such as those arising from failing to account for confounders or overtraining of models (Adedinsewo *et al.* 2022). Despite these advances in analytical methods, improving the standardisation and recording of female-specific risk factors and diagnoses in routine healthcare records remains essential for the effective prevention of ASCVD in females.

Another key aspect of commonly used cardiovascular risk estimation systems is their static nature. Most clinical practice guidelines recommend a cardiovascular risk assessment in individuals who are 40 years of age or older. With advancements in digitalisation and electronic health record systems, there is a potential to shift this dogma towards a dynamic risk estimation system that can be updated over time. This would allow for multiple risk assessments throughout a female’s life, such as during healthcare interactions such as pregnancy, enabling better targeting of young women who might otherwise be missed. However, we do feel it is essential to carefully consider the health benefits and costs of any risk assessment strategy recommended in practice.

### Challenges and future perspectives in research

The key recommendations and suggestions for next steps in cardiovascular health for females are provided in Table 4. It is important to consider the data used to develop cardiovascular risk estimation systems. All current risk estimation systems use observational data for development. Studies that include only participants from observational cohorts often introduce selection bias, as they typically involve the healthiest individuals from the least deprived areas. Using electronic healthcare records helps reduce selection bias, and there are methods available to address the main issue of missing data. Large, general population datasets based on healthcare records can improve representativeness, ensuring sufficient inclusion of younger females, ethnic minorities and female-specific risk factors in derivation cohorts.

**Table 4:** Key recommendations for future research in cardiovascular disease in females.

Recommendations	Details
Improve research practice	• Use routinely collected healthcare data to improve representatives rather than rely on participation in cohort studies • Consideration of socioeconomic status and ethnicity as standard practice. Improved education and coding practices • Inclusion of non-ASCVD in clinical endpoints and consistency of the definition of cardiovascular outcome
Focus on younger age groups	• Undertake studies specifically in younger females with longer follow up to capture events • Assess risk over life course starting from young adults • Evaluate sex and age interactions between the relationship with risk factors and ASCVD • Further examination of novel strategies for risk prediction in younger age groups (e.g. polygenic risk scores)
Improve research on female-specific factors	• Use of globally agreed definitions and nomenclature for gynaecological symptoms • Improve recording of symptoms in health services • Undertake more observational studies examining the association of gynaecology symptoms, conditions and management of gynaecological disorders and cardiovascular disease • Investigate the interaction of obesity with traditional and female-specific risk factors • Externally validate QR4 and assess the clinical added value of incorporating female-specific risk factors into risk prediction models
Examine risk in under-served groups and populations	• Create a study cohort of transgender people to follow over time • Improve understanding of the effects of hormonal medication in the transgender population • Work with low and middle income countries on improving data collection and management as well as other approaches that within their context that can be used to investigate cardiovascular female’s health

In addition, research practices need to be enhanced. Consistency in defining cardiovascular events, including non-ASCVD conditions, extending follow-up periods to capture risk in younger females and standardising the reporting of both absolute and relative risk will provide more robust evidence for the prevention of CVD in females. Steps are being undertaken to improve the recording of female-specific factors outside of pregnancy, such as the introduction of a reporting system for normal and abnormal uterine bleeding (Munro *et al.* 2018). Issues with ethnicity data are being highlighted, strengthening the importance of accurate coding of self-identified ethnicity being incorporated routinely into observational studies (Pineda-Moncusí *et al.* 2024). However, for these systems to add value, it is important to provide education to healthcare professionals and encourage consistent use of coding systems. In addition, natural language processing tools are being developed to identify uncoded data, which could enhance the use of healthcare record data, particularly in primary care. Novel techniques, such as machine learning approaches, may also be applied to incorporate additional markers such as cardiac biomarkers and multimodal imaging data.

In addition to female-specific factors, the complex relationship between obesity and cardiovascular risk factors in females requires further study. Obesity is not only associated with diabetes, hypertension and abnormal lipids, but also with female-specific risk factors such as pre-eclampsia, gestational diabetes, polycystic ovary syndrome and gynaecological cancers. Furthermore, the increased cardiovascular risk associated with obesity is observed across ethnicities. With the rising prevalence of obesity, particularly among younger populations (Sattar *et al.* 2023), advancing obesity research is crucial to reducing the burden of ASCVD in females.

Finally, it is important to understand cardiovascular risk in under-researched populations, in minority groups, the transgender population and also globally in low- and middle-income countries. The global burden of cardiovascular disease is increasingly shifting to low- and middle-income countries. Though observational data from countries such as China may be used to identify risk factors in these populations (Li *et al.* 2020), data-driven approaches used in high-income countries may not be directly applicable. Resource-limited countries may therefore need alternative strategies, and increased funding is needed to improve cardiovascular health in females in these regions.

## Conclusions

The development of CVD varies by sex and age, therefore cardiovascular risk estimation systems should incorporate both sex and age interactions with risk factors to improve ASCVD risk estimates. As the incidence of non-ASCVD in females continues to rise, it is crucial to adopt a more holistic approach to risk assessment that extends beyond the traditional ASCVD outcomes. The key risk factors for cardiovascular disease in females include traditional, female-prevalent disease, female-specific and social factors. Female-specific factors can be further divided into pregnancy- and non-pregnancy-related. There has been limited research for non-pregnancy-related factors such as heavy menstrual bleeding. This may be partly due to an insufficient number of younger females and follow-up periods which are not long enough to capture cardiovascular events. Furthermore, derivation cohorts used to derive the risk estimation systems often include older females, and most risk estimation systems can only be used in females from 30 to 40 years of age. QR4 is the only cardiovascular risk estimation system that can be used in young females from 18 years of age, which includes two female-specific risk factors. External validation of QR4 is essential, and further research is needed to evaluate the clinical added value of incorporating female-specific factors into cardiovascular risk estimation systems. Finally, alongside improving overall research practices and the coding of female-specific factors in healthcare records, future research must include populations from low- and middle-income countries, as well as smaller minority groups, to ensure improvement in the global prevention of cardiovascular disease in females.

## Declaration of interest

NS has consulted for and/or received speaker honoraria from Abbott Laboratories, AbbVie, Amgen, AstraZeneca, Boehringer Ingelheim, Eli Lilly, Hanmi Pharmaceuticals, Janssen, Menarini-Ricerche, Novartis, Novo Nordisk, Pfizer, Roche Diagnostics and Sanofi, and received grant support paid to his university by AstraZeneca, Boehringer Ingelheim, Novartis and Roche Diagnostics outside the submitted work. JAM has provided consultancy advice (all paid to institution; no personal remuneration) to Gedeon Richter. DMK has received honoraria from Roche Diagnostics and Abbott Diagnostics outside the submitted work. All other authors have no interests to declare.

## Funding

TA is supported by the Medical Research Councilhttps://doi.org/10.13039/501100000265, Precision Medicine Grant (MR/W006804/1). MdB and DMK are supported by an Intermediate Basic Science Research Fellowship and Research Excellence Award from the British Heart Foundationhttps://doi.org/10.13039/501100000274 (FS/IBSRF/23/25161, RE/24/130012). JAM received funding from Wellcome Fellowship 209589/Z/17/Z.

## Author contribution statement

TA, JAM and DMK conceived and designed this review. TA did the search and selected the studies for inclusion. TA drafted the manuscript and MdB, AA, NS, JAM and DMK revised it critically for important intellectual content. All authors approved the final manuscript.
